# Will women soon outperform men in open-water ultra-distance swimming in the ‘Maratona del Golfo Capri-Napoli’?

**DOI:** 10.1186/2193-1801-3-86

**Published:** 2014-02-13

**Authors:** Christoph Alexander Rüst, Romuald Lepers, Thomas Rosemann, Beat Knechtle

**Affiliations:** Institute of General Practice and for Health Services Research, University of Zurich, Zurich, Switzerland; INSERM U1093, Faculty of Sport Sciences, University of Burgundy, Dijon, France; Gesundheitszentrum St. Gallen, Vadianstrasse 26, 9001 St., Gallen, St. Gallen, Switzerland

**Keywords:** Sex difference, Swimmer, Woman, Man, Extreme endurance

## Abstract

This study investigated the change in sex differences across years in ultra-distance swimming performances at the 36-km ‘Maratona del Golfo Capri-Napoli’ race held from 1954 to 2013. Changes in swimming performance of 662 men and 228 women over the 59-year period were investigated using linear, non-linear and hierarchical regression analyses. Race times of the annual fastest swimmers decreased linearly for women from 731 min to 391 min (*r*^*2*^ = 0.60, *p* < 0.0001) and for men from 600 min to 373 min (*r*^*2*^ = 0.30, *p* < 0.0001). Race times of the annual top three swimmers decreased linearly between 1963 and 2013 for women from 736.8 ± 78.4 min to 396.6 ± 4.5 min (*r*^*2*^ = 0.58, *p* < 0.0001) and for men from 627.1 ± 34.5 min to 374.1 ± 0.3 min (*r*^*2*^ = 0.42, *p* < 0.0001). The sex difference in performance for the annual fastest decreased linearly from 39.2% (1955) to 4.7% (2013) (*r*^*2*^ = 0.33, *p* < 0.0001). For the annual three fastest competitors, the sex difference in performance decreased linearly from 38.2 ± 14.0% (1963) to 6.0 ± 1.0% (2013) (*r*^*2*^ = 0.43, *p* < 0.0001). In conclusion, ultra-distance swimmers improved their performance at the ‘Maratona del Golfo Capri-Napoli’ over the last ~60 years and the fastest women reduced the gap with the fastest men linearly from ~40% to ~5-6%. The linear change in both race times and sex differences may suggest that women will be able to achieve men’s performance or even to outperform men in the near future in an open-water ultra-distance swimming event such as the ‘Maratona del Golfo Capri-Napoli’.

## Introduction

An ultra-endurance competition is defined as an event of six hours in duration or longer (Zaryski and Smith 2005). Among the different ultra-endurance competitions held worldwide, open-water ultra-distance swimming is of increasing popularity. The number of open-water ultra-swim competitions continues to increase as well as the number of finishers in these races (http://www.openwaterswimming.com). For example, the participation in the ‘English Channel Swim’ increased in the last ten years exponentially for both women and men (Fischer et al. 2013). The number of swimmers per decade increased from the 1991–2000 period to the 2001–2010 period by 171% for men and by 135% for women, respectively (Eichenberger et al. 2012a). Similar findings have been observed in freshwater (Eichenberger et al. 2013) and indoor (Eichenberger et al. 2012b) ultra-endurance swimming, where the number of both participants and finishers increased in the last years.

The aspect of sex differences in ultra-endurance swimming performance has been recently investigated for indoor pool (Eichenberger et al. 2012b) and open-water ultra-distance swimmers (Eichenberger et al. 2012a; Fischer et al. 2013; Rüst et al. 2014; Vogt et al. 2013) and was leading to disparate findings. For recreational 12-hour indoor swimmers, Eichenberger et al. (2012b) showed that the performance of the annual fastest swimmers did not differ between women and men. Similarly, Eichenberger et al. (2012a) evidenced that the annual best performances at the 34-km ‘English Channel Swim’ were similar between men and women. In contrast, in an open-water ultra-endurance swim held in a freshwater lake covering ~26 km, the annual fastest men were on average ~11.5% faster than the annual fastest women (Eichenberger et al. 2013). However, recent studies investigating elite open-water ultra-distance swimmers showed different findings regarding the change in sex difference across years. In the 32-km ‘Traversée Internationale du Lac St-Jean’ the sex difference decreased between 1955 and 2012 from ~14% in 1973 to ~4% in 2012 (Rüst et al. 2014) and in the 10-km open-water races of FINA (Féderation Internationale de Natation), the sex difference remained unchanged at ~7% between 2008 and 2012 (Vogt et al. 2013).

These apparent disparate findings might be due to the length of the swim race, the different water conditions such as water temperature and the performance level of the participants. In the 34-km ‘English Channel Swim’, where the water temperature varied between 15°C and 18°C (http://www.channelswimmingassociation.com), the annual fastest women and men swam at a similar velocity (*i.e.* men 0.84 ± 0.18 m/s; women 0.89 ± 0.20 m/s) (Eichenberger et al. 2012a). In the indoor pool swim ‘Zurich 12 h Swim’ where the water temperature was kept constant at ~28°C, the annual fastest women and men also swam at a similar velocity (*i.e.* men 0.88 ± 0.06 m/s; women 0.79 ± 0.19 m/s) (Eichenberger et al. 2012b). In contrast, at the 26-km ‘Marathon Swim in Lake Zurich’ where the water temperature varied between 16.2°C and 25.9°C across years, the swimming performance of the annual fastest men was significantly faster (1.09 ± 0.10 m/s) compared to the annual fastest women (0.97 ± 0.07 m/s) (Eichenberger et al. 2013). Interestingly, it has also been shown that water temperature at the 26-km ‘Marathon Swim in Lake Zurich’ was significantly and negatively associated with swimming performance for the fastest swimmers independent of the sex (Eichenberger et al. 2013). Regarding the fitness level of the participants, recreational athletes were investigated in the 12-hour indoor pool swim (Eichenberger et al. 2012b), in the ‘Marathon Swim in Lake Zurich’ (Eichenberger et al. 2013) and in the ‘English Channel Swim’ (Fischer et al. 2013). The ‘English Channel Swim’ is in fact not a race where swimmers have to cross the Channel as solo swimmers. In contrast, elite swimmers were investigated in the ‘Traversée Internationale du Lac St-Jean’ (Rüst et al. 2014) and in the 10 km FINA competitions (Vogt et al. 2013).

Differences in swimming performance between women and men might be explained by differences in anthropometric characteristics between women and men such as body fat (Zuniga et al. 2011). Zuniga et al. (2011) showed for young sprint swimmers that body fatness was the only difference between female and male swimmers with no differences for other anthropometric characteristics such as musculoskeletal size, muscularity, skeletal size, total body mass, or body breadth dimensions. Regarding the influence of anthropometry on swimming efficiency, women have a smaller body size resulting in smaller body drag, a smaller body density with a greater body fat percent and shorter lower limbs, resulting in a more horizontal and streamlined position and therefore a smaller underwater torque (Lavoie and Montpetit 1986; Pendergast et al. 1977).

Apart from gender differences in body fat, swimming efficiency is also different between women and men. Buoyancy is higher in women through a lower underwater torque, which can be defined as the tendency for the feet to sink (Pendergast et al. 1977). In addition, and in contrast to running where the energy cost appeared similar between women and men, the energy cost of freestyle swimming has been shown to be significantly higher in men compared to women (Pate et al. 1987; Pendergast et al. 1977).

Apart of anthropometric characteristics, swimming economy should also be considered as an explanation for the sex difference. Swimming economy is considered as one of the most important predictors in swimming performance (Chatard et al. 1990; Fernandes and Vilas-Boas 2012; Smith et al. 2002). Three swimming economy related parameters are known such as the net energy cost corresponding to v VO_2_max (Cv VO_2_max), the slope of the regression line obtained from the energy expenditure (E) and corresponding velocities during an incremental test (C_slope_), and the ratio between the mean E value and the velocity mean value of the incremental test (C_inc_) (Fernandes et al. 2005). The investigation of the relationship between the time limit at the minimum velocity eliciting the individual's maximal oxygen consumption (TLim-v VO_2_max) and the above mentioned swimming economy related parameters showed that TLim-v VO_2_max seemed to depend in women more on swimming economy than in men (Fernandes et al. 2005).

Differences in the endocrine system between women and men may not account for performance differences in long-distance swimming. Dulac et al. (1987) showed that male and female swimmers had a similar metabolic and hormonal response to a long-distance swimming competition in cold water.

Changes in sex differences across years seemed to be different regarding the different sports disciplines. It has been reported sex differences in ultra-endurance performance decreased these last decades in specific sports disciplines such as running (Hoffman 2010) and multi-sports disciplines such as triathlon (Lepers 2008; Rüst et al. 2012a) although for running a plateau in the sex differences in running distances from 1,500 m to the marathon of ~11% between 1980 and 1996 has been shown (Sparling et al. 1998). For example, in 161-km ultra-marathon running, women have improved their performance between 1977 and the late 80’s but the sex differences in performance remained stable at ~20% in the past two decades (Hoffman 2010). Similar findings have been reported for ultra-distance triathletes competing in ‘Ironman Hawaii’ (Lepers 2008; Rüst et al. 2012a). From 1983 to 2012, the sex difference in overall race time decreased significantly from 15.2% to 11.3%. Regarding the different disciplines, the sex differences remained unchanged for swimming (~12.5%) and cycling (~12.5%) but decreased for running from 13.5% to 7.3% (Rüst et al. 2012a).

Based upon findings in ultra-marathon running (Hoffman 2010) and in ultra-distance triathlon (Rüst et al. 2012a), we hypothesized that the sex difference in open-water ultra-distance swimming performance would decrease across the years. To test this hypothesis, we examined the changes in sex difference in ultra-swim performance across the years over nearly 60 years from 1954 to 2013 at the 36-km ‘Maratona del Golfo Capri-Napoli’ swim race.

## Materials and methods

### Ethics

All procedures used in the study met the ethical standards of the Swiss Academy of Medical Sciences and were approved by the Institutional Review Board of Kanton St. Gallen, Switzerland, with a waiver of the requirement for informed consent of the participants given the fact that the study involved the analysis of publicly available data.

### The race

The ‘Maratona del Golfo Capri-Napoli’ is held annually since 1954 in mid-June. The route for the swimmers is 36 km long and it is divided into two parts. The first one is free for the swimmers and goes from ‘Marina Grande’ in Capri Island to ‘Napoli Promenade’, close to ‘Castel dell’Ovo’. The swimmers find a buoy where they have to turn on the left, leaving the buoy on the left. From this buoy, the route is obligatory where the swimmers have to cover the last kilometer of the race swimming following the coast until the arrival area which is located before the small beach of ‘Rotonda Diaz’. In mid-June, the mean water temperature of the Mediterranean Sea is at ~22°C (http://www.temperatureweather.com/mediterr/weather/en-weather-in-italy-napoli.htm).

### Data sampling and data analysis

All race results of all athletes who ever participated in the ‘Maratona del Golfo Capri-Napoli’ between 1954 and 2013 were analysed regarding the association of participation and performance. The data set for this study was obtained from the race website http://www.caprinapoli.com. All female and male solo swimmers during the 1954–2013 period were considered for data analysis. The races times of the annual top and of the annual top three women and men were retrieved from the race website http://www.caprinapoli.com. Due to the low number of successful annual finishers it was not possible to analyse a higher number of results for each year. The sex difference in swimming performance was calculated using the equation ([swim time in women] – [swim time in men])[swim time in men] × 100, where the sex difference were calculated for every pair of equally placed athletes (*e.g.* between female and male winner, between female and male 2nd place, etc*.*). The performance of the overall fastest, the overall three fastest and the overall ten fastest swim times ever for women and men were determined for the 59-year period and compared.

### Statistical analysis

In order to increase the reliability of the data analyses, each set of data was tested for normal distribution and for homogeneity of variances prior to statistical analyses. Normal distribution was tested using a D’Agostino and Pearson omnibus normality test and homogeneity of variances was tested using a Levene’s test. Trends in participation were analyzed using regression analyses with linear and exponential growth equation models. For each set of data (*e.g.* men and women), both models (*i.e.* linear versus exponential) were compared using Akaike’s Information Criteria (AICc) to determine the model with the highest probability of correctness. The AIC is a measure of the relative quality of a statistical model for a given set of data (Akaike 1974). It provides a means for model selection and deals with the trade-off between the goodness of fit of the model and the complexity of the model (Akaike 1974). Single and multi-level regression analyses investigated the changes in both performance and sex difference in performance of the fastest finishers. A hierarchical regression model avoided the impact of a cluster-effect on results when an athlete finished more than once for the analysis of the annual top or the annual top three athletes. Since the change in sex difference in endurance is assumed to be non-linear (Reinboud 2004), we additionally calculated the non-linear regression model that fits the data best. We compared then the linear with the best-fit non-linear models using AIC and F-test in order to show which model would be the most appropriate to explain the trend of the data. To find significant differences between swim times of the annual top and annual top three women and men and between the top three and the top ten ever, a Student’s *t*-test was used in case of normal distributed data with Welch’s correction in case of unequal variances and a Mann–Whitney test in case of not normal distributed data. To determine the performance of the fastest swimmers by origin, multiple pairings of countries were compared using one-way analysis of variance (ANOVA) with subsequent Sidak’s multiple comparison post-hoc test. Statistical analyses were performed using IBM SPSS Statistics (Version 21, IBM SPSS, Chicago, IL, USA) and GraphPad Prism (Version 6.01, GraphPad Software, La Jolla, CA, USA). Significance was accepted at *p* < 0.05 (two-sided for *t*-tests). Data in the text are given as mean ± standard deviation (SD).

## Results

### Participation trends

**Figure 1:**
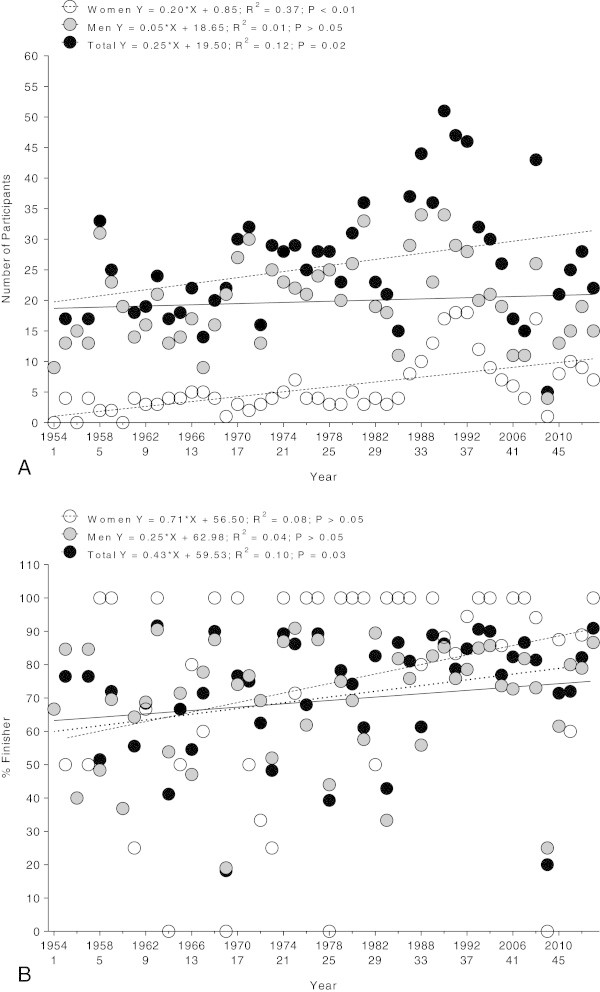
**Number (Panel A) and percent finisher rate (Panel B) of female, male and overall participants.**

**Figure 2:**
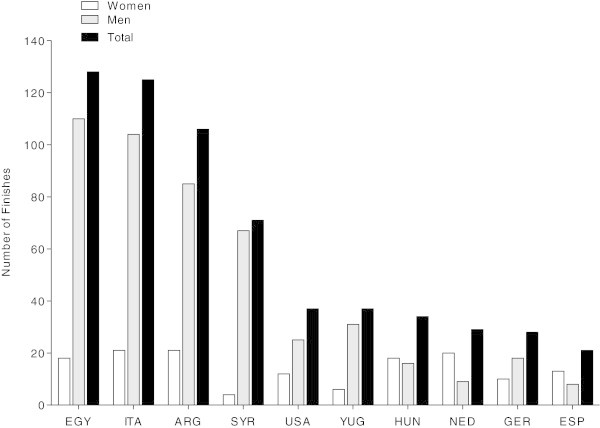
**Number of male, female and overall finishes per country.** EGY = Egypt, ITA = Italy, ARG = Argentina, SYR = Syria, USA = United States of America, YUG = Yugoslavia, HUN = Hungary, NED = Netherlands, GER = Germany, ESP = Spain.

Between 1954 and 2013, a total of 1,206 swimmers participated in the race. Among the 276 female starters, 228 (82.1%) finished and 48 (17.9%) did not finish the race. Among the 952 male starters, 662 (69.5%) finished while 290 (30.5%) were not able to finish. The annual number of female participants increased (*r*^*2*^ = 0.37, *p* < 0.01) whereas the annual number of male participants remained stable (*r*^*2*^ = 0.01, *p* > 0.05) over the years. The annual number of overall participants showed no change across years (Figure 1A). Among women, 56 swimmers finished the race more than once where Irene van der Laan has the record with nine finishes. Among men, 144 swimmers finished two times or more where Claudio Plit has the record with 15 finishes. The percentage of overall finishers increased across years (*r*^*2*^ = 0.03, *p* < 0.05), although the annual percentage of both female and male finishers showed no change across years (Figure 1B). Most of the finishers originated from Egypt (*i.e.* 110 men and 18 women), followed by finishers from Italy (*i.e.* 103 men and 19 women) and Argentina (*i.e.* 83 men and 20 women) (Figure 2).

### The fastest race times ever

**Figure 3:**
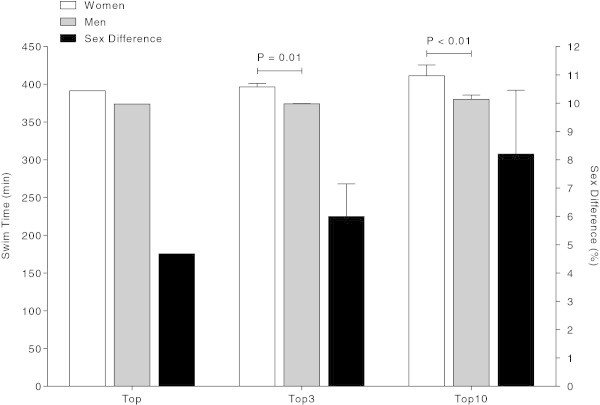
**Swim time and percent sex difference of the fastest swimmers ever, the three fastest swimmers ever and the ten fastest swimmers ever.**

The fastest men were always faster than the fastest women. The race records belong for men to Trent Grimsey (Australia) achieved in 2012 with 389.5 min and for women to Angela Maurer (Germany) in 2003 with 428.1 min (see top times in Figure 3). The sex difference in the race records is equal to 8%. The average race time of the three fastest swimmers ever was 425.6 ± 4.7 min for women and 392.3 ± 3.0 min for men, respectively, with a corresponding sex difference in performance of 8.5 ± 0.5%. The average race time of the ten fastest swimmers ever was 432.6 ± 6.5 min for women and 397.2 ± 3.9 min for men, respectively, with a corresponding sex difference in performance of 8.9 ± 0.8%.

### Performance trends across years

**Figure 4:**
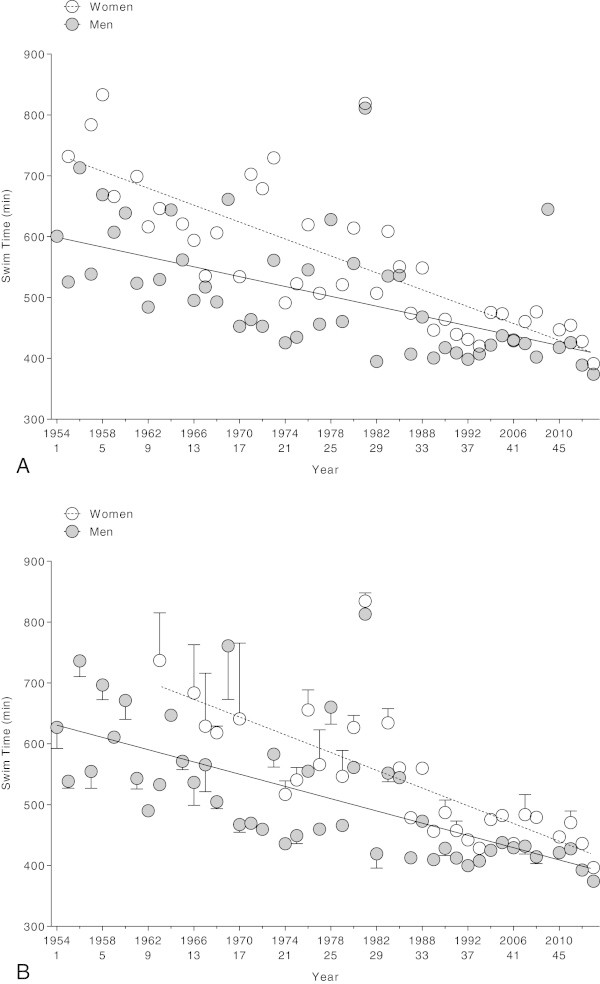
**Swim time of the annual top (Panel A) and annual top three (Panel B) women and men across years.**

Between 1954 and 2012, the swim time of the annual fastest women decreased from 731 min (1955) to 391 min (2013) (*r*^*2*^ = 0.60, *p* < 0.0001) and for the annual fastest men from 600 min (1954) to 373 min (2013) (*r*^*2*^ = 0.30, *p* < 0.0001) (Figure 4A). For the annual top three women, swim times decreased from 736.8 ± 78.4 min (1963) to 396.6 ± 4.51 min (2013) (*r*^*2*^ = 0.58, *p* < 0.0001) and for the annual top three men from 627.1 ± 34.5 min (1954) to 374.1 ± 0.3 min (2012) (*r*^*2*^ = 0.42, *p* < 0.0001) (Figure 4B). The inclusion of athletes with repeated finishes among the annual winners and the annual top three had no effect on the results (Table 1). The comparison of the linear versus the non-linear regression analyses showed a linear decrease for both the annual winners and the annual top three (Table 2).

**Table 1:** **Multi-level regression analyses for changes in swim times across years (Model 1) and with correction for multiple participations (Model 2) for the annual fastest and annual three fastest finishers**

Model	***β***	SE ( ***β*** )	Stand. ***β***	T	***P***
**Annual fastest men**					
**1**	−3.058	0.673	−0.556	−4.543	< 0.001
**2**	−3.058	0.673	−0.556	−4.543	< 0.001
**Annual three fastest men**					
**1**	−3.737	0.399	−0.620	−9.355	< 0.001
**2**	−3.737	0.399	−0.620	−9.355	< 0.001
**Annual fastest women**					
**1**	−5.197	0.671	−0.779	−7.747	< 0.001
**2**	−5.197	0.671	−0.779	−7.747	< 0.001
**Annual three fastest women**					
**1**	−5.766	0.423	−0.798	−13.621	< 0.001
**2**	−5.766	0.423	−0.798	−13.621	< 0.001

**Table 2:** **Comparison of linear and non-linear regression analysis of changes in swim time across years in the annual fastest and annual three fastest finishers to determine which model is the best**

Swim time	Kind of regression	Sum of squares	DOF	AICC	Best regression AIC-test	Best regression F-test	Delta	Probability	Likelihood
Annual fastest men	Polynomial	3120.08	42	432.8	Linear	Linear	7.5	0.02	97.8%
	Linear	3237.72	46	425.2					
Annual fastest women	Polynomial	1863.47	35	357.0	Linear	Polynomial	3.1	0.1	82.5%
	Linear	2183.96	39	353.9					
Annual three fastest men	Polynomial	3254.07	44	419.8	Linear	Linear	2.8	0.2	80.6%
	Linear	3208.71	45	417.0					
Annual three fastest women	Polynomial	1482.07	26	262.0	Linear	Linear	7.8	0.02	98.1%
	Linear	1337.63	28	254.2					

For all investigated regressions, linear was the best model. DOF = degrees of freedom, AIC = Akaike information criterion.

### Change in sex difference in performance over time

**Figure 5:**
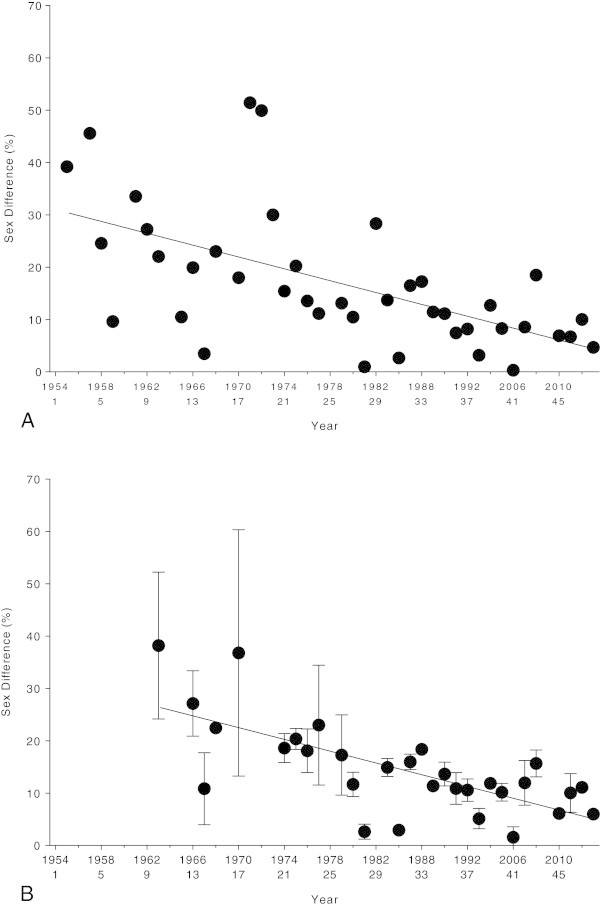
**Sex differences in performance of the annual top (Panel A) and annual top three (Panel B) women and men across years.**

The sex difference in performance of the annual fastest swimmers decreased from 39.2% (1955) to 4.7% (2013) (*r*^*2*^ = 0.33, *p* < 0.0001) (Figure 5). The sex difference in performance of the annual three fastest swimmers decreased from 38.2 ± 14.0% (1963) to 6.0 ± 1.0% (2013) (*r*^*2*^ = 0.43, *p* < 0.0001) (Figure 4B). The comparison of the linear versus the non-linear regression analyses showed a linear decrease in the sex difference for both women and men (Table 3).

**Table 3:** **Comparison of linear and non-linear regression analysis of changes in sex differences across years in the annual fastest and annual three fastest finishers to determine which model is the best**

Sex difference	Kind of regression	Sum of Squares	DOF	AICC	Best regression AIC-test	Best regression F-test	Delta	Probability	Likelihood
Annual fastest	Polynomial	3878.5	35	198.2	Linear	Linear	4.7	0.08	91.6%
	Linear	4364.0	39	193.4					
Annual three fastest	Polynomial	981.6	15	160.6	Linear	Linear	41.6	9.0 e^-10^	100%
	Linear	1474.6	28	118.9					

For all investigated regressions, linear was the best model. DOF = degrees of freedom, AIC = Akaike information criterion.

### Performance by origin of the athletes

**Figure 6:**
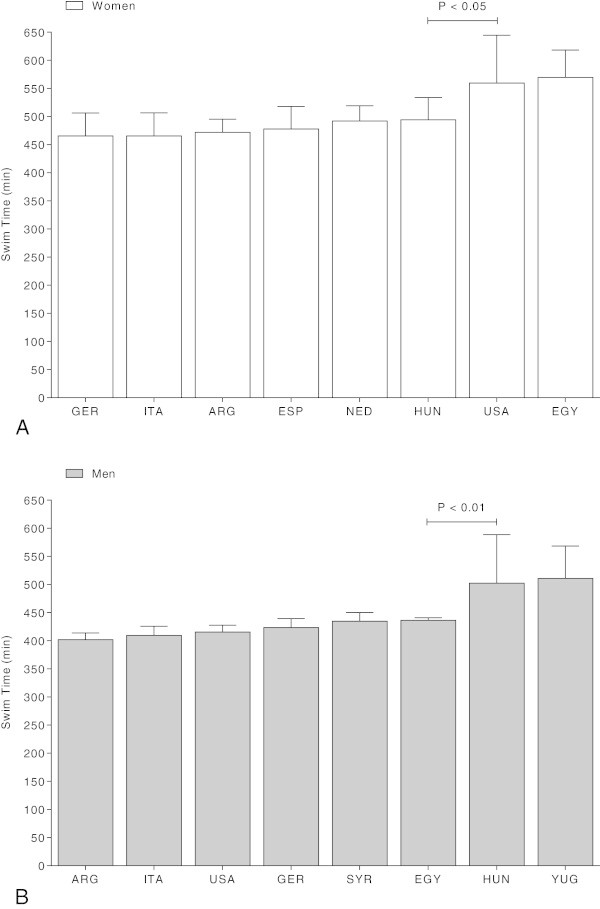
**Performance of the top ten ever women (Panel A) and men (Panel B) per country for countries where at least ten men or women finished the race successfully.** *P*-value is given in case of significant differences between adjacent countries. EGY = Egypt, ITA = Italy, ARG = Argentina, SYR = Syria, USA = United States of America, YUG = Yugoslavia, HUN = Hungary, NED = Netherlands, GER = Germany, AUS = Australia.

Considering the performance of the swimmers regarding their origin (Figure 6), the fastest female swim times were achieved by German swimmers (465.4 ± 40.8 min), followed by swimmers from Italy (472.0 ± 23.7 min) and Argentina (489.6 ± 27.8 min). The fastest male swimmers originated from Argentina (409.5 ± 8.7 min) followed by swimmers from Italy (414.5 ± 12.0 min) and the United States of America (415.5 ± 12.2 min) (Figure 6).

## Discussion

The aim of this study was to investigate the change in sex difference in performance in the 36-km ‘Maratona del Golfo Capri-Napoli’ from 1954 to 2013. The swim times decreased linearly over the studied period for both men and women, and the sex differences in performance decreased linearly for both the annual winners from 1955 to 2012 and the annual top three finishers from 1963 to 2012. The linear change in both swim times and sex differences may suggest that women will be able to achieve men’s performance or even to outperform men in ‘Maratona del Golfo Capri-Napoli’ in the near future.

It has been reported that the sex difference in sports performance is non-linear (Reinboud 2004). However, in the present ultra-swimmers, the changes in both swim times and sex differences in performance were linear, but not non-linear. The linear decrease in sex difference in ultra-swimming performance observed in the present study differs from previous findings for triathlon and in ultra-distance running races (Hoffman 2010; Lepers 2008; Rüst et al. 2012b; Sigg et al. 2013). Indeed, it has been shown that for triathlon (Lepers 2008; Rüst et al. 2012b; Sigg et al. 2013) and ultra-running (Hoffman 2010), the sex difference in performance plateaued or even increased these last decades. An analysis of performance in Ironman triathletes competing in ‘Ironman Hawaii’ between 1981 and 2007 showed that overall race times decreased rapidly from 1981 but remained stable since the late 1980s (Lepers 2008). Between 1988 and 2007, the sex difference remained stable for swimming (+0.1% per decade), increased for cycling (+0.8% per decade), and decreased for running (−2.8% per decade). The sex difference in performance for overall Ironman race time remained stable in the last two decades (−0.5% per decade) (Lepers 2008). In longer triathlon distances than the Ironman distance, the sex differences remained unchanged or even increased across years. For example, in Double Iron ultra-triathlons held between 1985 and 2012, the sex difference remained unchanged for overall race times (27.1%), the swim split times (26.8%), the cycling split times (25.2%), and the run split times (32.4%) (Sigg et al. 2013). In Triple Iron ultra-triathlon, the sex differences in overall race times increased from 10% in 1992 to 42% in 2011 while the sex differences decreased in swimming split times (from 35% to 28%), but increased in running (from 12% to 40%) and cycling (from 10% to 64%) split times (Rüst et al. 2012b). In ultra-marathoners competing in 161-km ultra-marathons in North America the sex difference in performance was ~40% in 1977 and decreased to ~20% in 1989 with no further change until 2008 (Hoffman 2010).

A time period of two or three decades for the determination of a potential change in sex differences in open-water ultra-distance swimming performance was probably too short in previous investigations (Eichenberger et al. [Bibr CR9][Bibr CR10][Bibr CR11] Fischer et al. 2013 Vogt et al. 2013). In the ‘Marathon Swim Lake Zurich, a time period of 24 years (1987–2011) was investigated (Eichenberger et al. 2013). The sex difference in performance for female and male winners was on average ~11.5% with no change across years. In the ‘Zurich 12 h Swim’ athletes competing between 1996 and 2010 (14 years) were considered (Eichenberger et al. 2012b). The annual best performance was not different between women and men. In the ‘English Channel Swim’, data were available since 1875, however, only a time period of 36 years from 1975 to 2011 could be considered to investigate the sex differences between the annual fastest three women and men. For the mean of the annual fastest swim times, performances between women and men showed no differences (Eichenberger et al. 2012a). When the annual three fastest women and men were considered, the sex difference remained unchanged at 12.5 ± 9.6% over time (Fischer et al. 2013).

The progressive decrease in sex difference in performance in these ultra-swimmers over the 59-year period might be due to several reasons. Changes in training methods (Costa et al. 2013) and nutrition (Slattery et al. 2012) these last years might have improved performance. However, both women and men might have benefit from these improvements. Changes in anthropometric characteristics of ultra swimmers across years might also have an influence. It has been shown that anthropometric characteristics were related to performance in open-water ultra-swimming (Knechtle et al. 2010). Across years, top swimmers improved performance in 100 m pool-swimming between 1912 and 2008 (Charles and Bejan 2009). In the same period, body height and body slenderness increased. Swimming speeds increased in proportion to body height (Charles and Bejan 2009). Van Heest et al. (2004) reported that elite open-water swimmers were smaller and lighter than competitive pool swimmers. Since competitive open-water ultra-distance swimming is a rather young discipline (http://www.fina.org), athletes with specific anthropometric characteristics will most probably move from pool swimming to open-water swimming.

Another explanation for the decrease in sex difference might be drafting during open-water swimming. In triathlon (Landers et al. 2008) and in open-water ultra-distance swimming (Rüst et al. 2014), athletes draft one behind the other. Therefore, the fastest women may draft behind the fastest men and reduce drag (Chatard and Wilson 2003). Drafting may save energy since swimming behind another swimmers reduced oxygen uptake, heart rate, blood lactate and stroke rate.

The sex difference in performance reached in 2013 a value of 4.7% for the annual winners and of 6.0 ± 1.0% for the annual three fastest finishers. By comparison, for ultra-endurance cyclists competing in the ‘Race Across America’ between 1982–2012, the fastest men were 14-15% faster than the fastest women and the sex difference was ~25% for the annual three fastest woman and men in the last 30 years (Rüst et al. 2013). In running, the sex difference was at ~11-12% when considering running distances from 100 m to 200 km (Coast et al. 2004; Sparling et al. 1998). In 161-km trail running, the sex difference was even at ~20% (Hoffman 2010). The sex difference in running performance appears biological in origin (Cheuvront et al. 2005). Success in distance running is determined largely by aerobic capacity and muscular strength. Men have a larger aerobic capacity and greater muscular strength suggesting that the gap in running performances between men and women is unlikely to narrow naturally (Cheuvront et al. 2005). This might be true for running and ultra-running, however, not for ultra-swimming in cold water. Female open-water ultra-swimmers have a higher percent body fat compared to male open-water ultra-swimmers (Weitkunat et al. 2012). The higher body fat might be an advantage in longer ultra-distance swims (> 40 km) and at temperatures of < 20°C since body fat was not predictive in a fresh-water swim of ~27 km at water temperatures of ~20-22°C (Knechtle et al. 2010).

Apart from anthropometric characteristics, women improved freestyle swimming performance across years when the race results of male and female finalists in each freestyle event (*i.e.* 50 m, 100 m, 200 m, 400 m, 800 m, 1500 m) held at the Olympic Games between 1896 and 2008 were investigated (Stanula et al. 2012). Moreover, the difference between women and men became smaller with increasing race distance and women improved their performance more dynamically compared to men (Stanula et al. 2012). The increase in female swimming performance across years at the level of the Olympic Games was most probably due to the fact that women were less well trained than men during earlier years (Stefani 2006). A study investigating butterfly swimmers of different skills showed that differences between skill levels were related to the capacity of elite swimmers to assume a more streamlined position of trunk, head and upper limbs during leg actions, adopt a shorter glide and higher stroke rate to overcome great forward resistance, and generate higher forces and use better technique during the arm pull (Seifert et al. 2008).

Basic motor abilities might also be different in women and men. Pavić et al. (2008) investigated differences in basic motor abilities in young swimmers. The discriminative analysis of motor variables between female and male young swimmers showed that male subjects were superior in explosive strength, throw strength in particular, coordination and aerobic endurance, whereas female subjects showed a better performance in flexibility and movement frequency, leg movement in particular. In male young swimmers, the motor system was found to integrate coordination/agility, aerobic endurance and explosive strength, whereas in female young swimmers it integrated coordination in terms of cortical movement regulation, aerobic endurance, explosive strength and psychomotor speed (Pavić et al. 2008). These basic motor abilities might be differently developed into adulthood.

An interesting finding was that most of the finishers originated from Egypt for both women and men. The fastest swim times were achieved, however, by women originating from Germany and men originating from Argentina. In the 10-km races of the FINA, most of the participants originated from Brazil, followed by swimmers from Germany and Russia (Vogt et al. 2013). At the ‘English Channel Swim’, most of the competitors originated from England, most probably due to vicinity of the Channel for British swimmers (Eichenberger et al. 2012a). The best annual swim times were achieved by British swimmers. In the 10-km races of the FINA from 2008 to 2012 held all over the world, the athletes started most probably in the races held in their country or near to their country (Vogt et al. 2013). Although both the ‘English Channel Swim’ and the ‘Maratona del Golfo Capri-Napoli’ are of about the same distance, athletes of different nations participate in these races although both races were held in Europe. A potential explanation for these disparate findings could be the water temperature. British swimmers might be more familiar with the cold water in the ‘English Channel Swim’. The ‘Maratona del Golfo Capri-Napoli’ is held in the Mediterranean Sea with higher water temperatures. This might explain why Egypt swimmers were the most frequent finishers and German women and Argentinian men dominated the race.

### Strength, limitations and implications for future research

A strength of the present study is the long period of investigation of ~60 years and the comparison of linear versus non-linear changes of both swimming performances and sex differences over time. Limitations are that anthropometric (Knechtle et al. 2010) and training (Knechtle et al. 2010) characteristics of the swimmers are not recorded. It has been shown that the world’s fastest 100 m swimmers became taller and heavier (Charles and Bejan 2009) and both body height and body mass index were predictive for race time in open-water ultra-swimmers (Knechtle et al. 2010). Future studies need to investigate changes in anthropometric characteristics in ultra-distance open-water swimmers over time.

## Conclusion

In conclusion, the sex difference in swimming performance at the 36-km ‘Maratona del Golfo Capri-Napoli’ decreased in the last ~60 years to reach ~5-6% in 2013. The question whether women might reduce the gap with men in open-water ultra-swimming in the future could be raised. The linear change in both swim times and sex differences may suggest that women will be able to achieve men’s performance or even to outperform men in the near future in an open-water ultra-distance swimming event such as the ‘Maratona del Golfo Capri-Napoli’. Future studies need to investigate the changes in sex differences in longer swim distances and in extreme environment such as cold water. The female anthropometry with a higher body fat might be of advance in very long open-water ultra-swims (> 40 km) in cold water (<20°C).
